# Editorial for the Special Issue “Cytokines in Inflammatory Signaling”

**DOI:** 10.3390/ijms25115799

**Published:** 2024-05-26

**Authors:** Wai Po Chong

**Affiliations:** 1 School of Chinese Medicine, Hong Kong Baptist University, Hong Kong, China; chongwp@hkbu.edu.hk; Tel.: +852-3411-2928; 2 Institute for Research and Continuing Education, Hong Kong Baptist University, Shenzhen 518057, China

## 1. Introduction

The intricate interplay of cytokines within the inflammatory response serves as a cornerstone of both the maintenance of physiological homeostasis and the pathogenesis of proinflammatory diseases. The Special Issue “Cytokines in Inflammatory Signaling” presents a compelling collection of research articles that together paint a vivid picture of the multifaceted roles cytokines play in health and disease. 

At the forefront of this Special Issue is the exploration of neuroprotective strategies against central nervous system (CNS) disorders. Lee et. al. studied the effect of Sophoraflavanone G on the inhibition of MMP-9 expression in brain microvascular endothelial cells as a novel strategy for mitigating neuroinflammation, a common thread in neurodegenerative diseases [1]. Their study elegantly demonstrates the potential for phytochemicals to disrupt the deleterious cascade of inflammatory events triggered by TNF-α, highlighting a promising therapeutic pathway. The theme of protective mechanisms extends into the aquatic realm; another contribution to this Special Issue developed a zebrafish model elucidating the role of IL-6 in oxidative stress. IL-6 deficiency attenuated liver injury following Aeromonas hydrophila infection [2]. The study not only advances our understanding of IL-6’s function in the context of liver injury but also of its pathogenic role in promoting oxidative stress.

This Special Issue also features two studies that delve into the effects of novel peptides derived from proteinase inhibitors on asthma–chronic obstructive pulmonary disease (COPD) overlap (ACO) [3,4]. By showcasing the anti-inflammatory and antioxidant capabilities of these peptides, these articles provide new insights into novel therapeutic approaches for patients suffering from these debilitating respiratory conditions. The comparison to corticosteroid treatments invites a re-evaluation of current therapeutic strategies and encourages the pursuit of alternative and potentially more efficacious options.

A particularly intriguing facet of this Special Issue is the emphasis on sex differences in cytokine signaling pathways. The study on Sphingosine-1-phosphate (S1P) levels in lung physiology and tumor conditions reveals a differential regulatory landscape that could inform sex-specific therapeutic interventions [5]. This finding not only contributes to the broader narrative of personalized medicine but also raises important questions about the intersection of sex, disease, and cytokine activity.

The aging process and its relationship with cytokine activity is another critical area addressed in this Special Issue. The correlation between the anti-inflammatory Klotho protein and IFN-γ expression in the context of human aging offers valuable insights into the immune system’s adaptation over a lifetime [6]. This research study has the potential to inform interventions aimed at enhancing healthy aging and managing age-related diseases.

In the realm of adaptive immunity, the role of IL-38 in B cell biology and its paradoxical effects on plasma cell generation and antibody production are revealed in another contribution to this Special Issue [7]. The nuanced understanding of IL-38’s function provided by this study is a testament to the complex nature of cytokine signaling and its impact on the immune response.

This Special Issue also comprehensively elucidates the broader systemic effects of cytokines, as evidenced by two reviews: one on adipokines in the musculoskeletal and cardiovascular systems and one on the potential of cytokines as biomarkers for physical exercise [8,9]. These articles not only further our appreciation of the systemic nature of cytokine activity but also emphasize the translational potential of cytokine research in enhancing athletic performance and managing chronic diseases. Lastly, the brief report on IL-1 signaling in antiviral T cell immunity challenges us to reconsider the role of IL-1 in the context of infectious diseases and vaccination strategies [10]. This piece, in particular, underscores the delicate balance between cytokine-mediated protection and pathology, a recurring theme throughout this Special Issue.

In conclusion, the articles within this Special Issue collectively underscore the dynamic and often dualistic roles of cytokines in inflammatory signaling. From neuroprotection to aging, and from systemic health to targeted immune responses, the research presented here not only advances our scientific knowledge but also offers tangible hope for the development of novel therapeutic interventions. As we continue to unravel the complex tapestry of cytokines within the inflammatory response, the potential for significant clinical advancements remains both promising and profound.

## References

[1] Lee T.H., Chen J.L., Tsai M.M., Wu Y.H., Tseng H.C., Cheng L.C., Shanmugam V., Hsieh H.L. (2023). Protective Effects of Sophoraflavanone G by Inhibiting TNF-alpha-Induced MMP-9-Mediated Events in Brain Microvascular Endothelial Cells. Int. J. Mol. Sci..

[2] Zhai W., Wang Z., Ye C., Ke L., Wang H., Liu H. (2023). IL-6 Mutation Attenuates Liver Injury Caused by Aeromonas hydrophila Infection by Reducing Oxidative Stress in Zebrafish. Int. J. Mol. Sci..

[3] Silva L., Barbosa J.A.S., Joao J., Fukuzaki S., Camargo L.D.N., Dos Santos T.M., Campos E.C., Costa A.S., Saraiva-Romanholo B.M., Bezerra S.K.M. (2023). Effects of a Peptide Derived from the Primary Sequence of a Kallikrein Inhibitor Isolated from Bauhinia bauhinioides (pep-BbKI) in an Asthma-COPD Overlap (ACO) Model. Int. J. Mol. Sci..

[4] Barbosa J.A.S., da Silva L.L.S., Joao J., de Campos E.C., Fukuzaki S., Camargo L.D.N., Dos Santos T.M., Dos Santos H.T., Bezerra S.K.M., Saraiva-Romanholo B.M. (2023). Investigating the Effects of a New Peptide, Derived from the Enterolobium contortisiliquum Proteinase Inhibitor (EcTI), on Inflammation, Remodeling, and Oxidative Stress in an Experimental Mouse Model of Asthma-Chronic Obstructive Pulmonary Disease Overlap (ACO). Int. J. Mol. Sci..

[5] Terlizzi M., Colarusso C., Ferraro G., Falanga A., Monti M.C., Somma P., De Rosa I., Panico L., Pinto A., Sorrentino R. (2023). Sex Differences in Sphingosine-1-Phosphate Levels Are Dependent on Ceramide Synthase 1 and Ceramidase in Lung Physiology and Tumor Conditions. Int. J. Mol. Sci..

[6] Kaszubowska L., Foerster J., Kaczor J.J., Karnia M.J., Kmiec Z. (2023). Anti-Inflammatory Klotho Protein Serum Concentration Correlates with Interferon Gamma Expression Related to the Cellular Activity of Both NKT-like and T Cells in the Process of Human Aging. Int. J. Mol. Sci..

[7] Huard A., Wilmes C., Kiprina A., Netzer C., Palmer G., Brune B., Weigert A. (2023). Cell Intrinsic IL-38 Affects B Cell Differentiation and Antibody Production. Int. J. Mol. Sci..

[8] Sierawska O., Sawczuk M. (2023). Interaction between Selected Adipokines and Musculoskeletal and Cardiovascular Systems: A Review of Current Knowledge. Int. J. Mol. Sci..

[9] Malkowska P., Sawczuk M. (2023). Cytokines as Biomarkers for Evaluating Physical Exercise in Trained and Non-Trained Individuals: A Narrative Review. Int. J. Mol. Sci..

[10] Van Den Eeckhout B., Ballegeer M., De Clercq J., Burg E., Saelens X., Vandekerckhove L., Gerlo S. (2023). Rethinking IL-1 Antagonism in Respiratory Viral Infections: A Role for IL-1 Signaling in the Development of Antiviral T Cell Immunity. Int. J. Mol. Sci..

